# Rosmarinic acid protects against experimental diabetes with cerebral ischemia: relation to inflammation response

**DOI:** 10.1186/1742-2094-10-28

**Published:** 2013-02-17

**Authors:** Haiyun Luan, Zechun Kan, Yong Xu, Changjun Lv, Wanglin Jiang

**Affiliations:** 1 School of Pharmaceutical Sciences and Institute of Material Medica, Binzhou Medical University, Yantai, 264003, People's Republic of China

**Keywords:** RA, Cerebral ischemia, Diabetes, Nuclear factor kappaB, Inflammatory response, High-mobility group box1

## Abstract

**Background:**

Inflammatory activation plays a vital role in the pathophysiological mechanisms of stroke, exerting deleterious effects on the progression of tissue damage and may lead to the vascular damage in diabetes. The objectives of this study were to determine the effects of rosmarinic acid (RA) on a cultured neuronal cell line, SH-SY5Y *in vitro* and experimental ischemic diabetic stroke *in vivo*.

**Methods:**

For oxygen-glucose deprivation (OGD) and tumor necrosis factor-α (TNF-α) stimulated SH-SY5Y cell line *in vitro*, SH-SY5Y cells were incubated with RA. For an *in vivo* experiment, diabetic rats were subjected to middle cerebral artery occlusion (MACO) for 40 minutes followed by reperfusion for 23 h.

**Results:**

Treatment of SH-SY5Y cells with RA reduced the OGD-induced apoptosis and cytotoxicity, blocked TNF-α-induced nuclear transcription factor κB (NF-κB) activation, and decreased high-mobility group box1 (HMGB1) expression. At doses higher than 50 mg/kg, RA produced a significant neuroprotective potential in rats with ischemia and reperfusion (I/R). RA (50 mg/kg) demonstrated significant neuroprotective activity even after delayed administration at 1 h, 3 h and 5 h after I/R. RA 50 mg/kg attenuated histopathological damage, decreased brain edema, inhibited NF-κB activation and reduced HMGB1 expression.

**Conclusion:**

These data show that RA protects the brain against I/R injury with a favorable therapeutic time-window by alleviating diabetic cerebral I/R injury and attenuating blood–brain barrier (BBB) breakdown, and its protective effects may involve HMGB1 and the NF-κB signaling pathway.

## Background

Ischemic stroke is a leading cause of death and disability, resulting from a transient or permanent reduction in cerebral blood flow in a major brain artery. Each year 750,000 strokes occur in the US. A large number of survivors experience permanent neurological damage, which impacts the quality of life. The annual cost for the treatment of stroke victims is over 50 billion dollars per year. In spite of substantial research and development efforts, no sufficiently effective therapy for this obstinate illness is available.

Diabetes mellitus (DM) is associated with both microvascular and macrovascular diseases affecting several organs. Consequently, DM not only increases the risk of ischemic stroke but also increases recurrence of stroke and poorer outcomes after stroke [1,2]. For nearly 20 years, the number of Chinese diabetes patients has been in a growth spurt [3]. Therefore, investigation of the neuroprotective effects of diabetic stroke is warranted in non-streptozotocin (WT) middle cerebral artery occlusion (MCAO) rats.

The inflammatory response to brain injury, especially NF-κB activation, plays a vital role in the pathogenesis of ischemic stroke [4]; inhibition of NF-κB may represent a treatment strategy in ischemic stroke [5]. High-mobility group box1 (HMGB1), a nonhistone DNA-binding protein, has been reported to be massively released into the extracellular space immediately after an ischemic insult and to induce neuroinflammation and microglial activation in the post-ischemic brain [6]. Extracellular HMGB1 contributes to the pathogenesis of brain disorders. A large body of information indicates that HMGB1 is involved in severe inflammatory reactions and plays a significant part in the progression of ischemic brain injury [7-9]. Thus, HMGB1 is a key regulator of the neuro-immune system which is involved in progression of neurologic disorders. Therefore, inhibition of HMGB1 release may be a novel therapeutic strategy for treating diabetic cerebral ischemia.

Rosmarinic acid (RA) is a natural, phenol, antioxidant carboxylic acid. Many bioactivities of RA have been reported, such as anti-liver fibrosis [10], antisepsis [11] and anti-diabetic nephropathy [12]. There is no report which has demonstrated the role of HMGB1 and NF-κB in the neuroprotective effect of RA on diabetic rats with cerebral ischemic injury. In the present study, we, therefore, investigate the hypothesis that RA plays a neuroprotective role through the abrogation of HMGB1 release and NF-κB activation triggered by tumor necrosis factor-α (TNF-α) and ameliorates diabetic rats with MCAO-induced cerebral ischemia/reperfusion injury.

## Methods

### Materials

Rosmarinic acid (RA, Molecular Formula: C_21_H_20_O_10_; CAS NO.: 32769-01-0, purity >98.3%,) was provided by the State Key Laboratory of Long-acting Extended-Release and Targeting Drug Delivery System, Yantai, PR China.

### Cell culture

Human neuroblastoma (SH-SY5Y) cells were obtained from the Shanghai cell bank of the Chinese Academy of Sciences. SH-SY5Y cells were cultured and maintained in F12 + DMEM (1:1, v/v) media, supplemented with 10% FBS and 1% penicillin/streptomycin. Cells were kept at 37°C in a humidified 5% CO_2_/95% O_2_ incubator. The dissociated cells were seeded in poly-L-lysine-coated plates at a density of 5 × 10^5^/cm^2^ and cultured in DMEM (Shanghai lifetechnologies, PR China), supplemented with 10% (v/v) horse serum, 5% (v/v) fetal bovine serum, 100 U/ml penicillin and 0.1 mg/ml streptomycin. The fresh medium was changed twice weekly. After eight days of culture, the SH-SY5Y cells were randomly divided into seven groups: a normal group (no oxygen-glucose deprivation), an oxygen-glucose deprivation group (control group) and oxygen-glucose deprivation + RA groups (1, 3, 9, 27 and 81 μM). RA was dissolved in saline. For oxygen-glucose deprivation, the SH-SY5Y cells were washed twice in glucose-free balanced salt solution (BSS) containing 130 mM NaCl, 5.5 mM KCl, 1.8 mM CaCl_2_, 1.0 mM MgCl_2_, and 20 mM HEPES (pH 7.4), and incubated in BSS (no glucose) in a humidified chamber filled with 95% N_2_/5% CO_2_ for 3 h at 36.5°C. After oxygen-glucose deprivation, the cultures were replaced into neurobasal medium and incubated with RA (1, 3, 9, 27 and 81 μM) in a CO_2_ incubator for 12 h.

### Determination of cell viability, lactic dehydrogenase (LDH) leakage and apoptosis

After oxygen-glucose deprivation for 3 h followed by 12 h incubation with or without RA, cell viability was assessed using a 3-(4, 5)-dimethylthiahiazo (−z-y1)-3, 5-di-phenytetrazoliumromide (MTT) assay. LDH, an indicator of cell injury, was detected according to the description of the LDH assay kit (Beijing Zhongsheng Bioreagent, Beijing, PR China). LDH leakage rate (%) = A_e_/A_t_*100%. A_e_ indicated extracellular lactic dehydrogenase (LDH, cells culture fluid), A_t_ indicated intracellular and extracellular LDH (cells lysate). Apoptotic cells were evaluated using an Annexin-V fluorescein isothiocyanate (FITC) apoptosis detection kit (Haimen Beyotime Biotechnology Institute, Jiangsu, PR China. In brief, cells were harvested, washed and incubated at 4°C for 30 minutes in the dark with annexin-V FITC and propidium iodide, then analyzed on a FACS Vantage SE flow cytometer (Beckman Coulter, Fullerton, CA, USA).

### Animals

Adult male Sprague–Dawley rats were obtained from Shandong Luye Pharmaceutical Company (Yantai, PR China). All animals were housed individually at 22 ± 2°C and a relative humidity of 50 ± 10% with a 12-h light/dark cycle and had free access to chow and water. All procedures (including rat cerebral ischemia study protocol) were carried out in accordance with the National Institute of Health Guide for the Care and Use of Laboratory Animals (NIH Publications No. 80–23) revised 1996.

### Streptozotocin (*STZ*)-*induced diabetes animal model and rat cerebral ischemia study protocol*

Two hundred and eighty rats (fasting for 20 h) were induced by single intraperitoneal injection (*i*.*p*.) of STZ at a dose of 45 mg/kg. STZ was diluted in citrate buffer 0.1 M (pH 4.0). After STZ injection for three weeks, rats with a glycemia value between 11.0 to 22.0 mM were used. The diabetic rats were anesthetized with chloral hydrate (350 mg/kg, *i*.*p*.). Rectal temperature was recorded and maintained at 37°C throughout the surgical procedure. The operation of MCAO was carried out according to previous procedures with minor modifications [13]. The adequacy of the anesthesia was monitored by the level and stability of the mean arterial pressure (MAP) and absence of corneal reflex, and adequate levels of anesthesia and analgesia were ensured with supplemental i.p. injection of pentobarbital sodium given as required. The left common carotid artery was occluded, and the branches of the external carotid artery were dissected and divided. The internal carotid artery was followed rostrally and a 4–0 filament (Beijing Shadong Biology Company, Beijing PR China. A filament (with a diameter of 0.25, and a tip diameter of 0.34 mm to create a globular stopper) was introduced into the internal carotid artery and advanced until resistance was felt. The filament was removed after 40 minutes. The rats were kept under control temperature (24°C to 25°C) conditions for the first 24 h after surgery.

For dose–response studies, 70 rats were randomly divided into seven groups of 10 rats each plus 10 rats as control (no diabetic). RA at doses of 0, 12.5, 25, 50, 100 or 200 mg/kg was administered by intravenous bolus injection into the tail vein 30 minutes after reperfusion. Diabetic or vehicle-treated rats were administered with saline. Rectal temperature was determined once every 3 h for a total of eight times. Neurological deficits were determined at 24 h after ischemia followed by brain infarct volume examination.

For therapeutic time-window studies, 50 rats were randomly divided into five groups of 10 rats each. Rats received a dose of 50 mg/kg by intravenous bolus injection into the tail vein 1 h, 3 h, 5 h and 7 h after reperfusion. Vehicle-treated rats were administered with saline. Neurological deficits were determined 24 h after ischemia followed by brain infarct examination.

For anti-inflammatory mechanism studies, 45 rats were randomly divided into three subgroups of 15 rats each, plus 15 rats as control (no diabetic). Rats received doses of 50 mg/kg intravenous bolus injections into the tail vein 30 minutes after reperfusion. Diabetic or vehicle-treated rats were administered with saline. The above three groups were evaluated for Evans blue extravasation, Western blots analysis, and histopathological damage by NeuN staining.

For long-term studies, 30 rats were randomly divided into two groups of 15 rats each. Rats received doses of 50 mg/kg by intravenous bolus injection into the tail vein 30 minutes after reperfusion. The vehicle-treated rats were administered with saline. Neurological deficits were determined at the 3rd, 7th and 14th day after I/R. Fourteen days after I/R, seven rats in each group were randomly selected for brain infarct examination according to a previous method [14].

### Evaluation of neurological deficits

Neurological deficits were evaluated using a modified six-point scoring method [15] by an investigator who was blinded to each experimental group. The scale is 0, no neurological deficits (normal); 1, failure to extend left forepaw fully (mild); 2, circling to the left (moderate); 3, falling to the left (severe); 4, no spontaneous walking with a depressed level of consciousness (very severe), and 5, death.

### Evaluation of infarct volume and brain water content

After 23 h of reperfusion, rats were anesthetized with sodium pentobarbital (40 mg/kg) through intraperitoneal injection, and the brain was quickly removed. Total wet weight of the brain was measured accurately. Brain was cut at the forebrain 3 mm, and each brain was sliced into five coronal sections of 2-mm thickness each, then stained with a 2% solution of tetrazolium chloride (TTC, Sigma, Shanghai, PR China) in saline at 37°C for 20 minutes, and photographed. The images were digitized, and infarct volume was calculated with a Compix system computer (C imaging 1280 system, Compix Inc., Cranberry. Township, PA, USA). Afterwards, brain water content was determined as an indicator of cerebral edema using a wet/dry method.

### Evaluation of blood–brain barrier (BBB) leakage with Evans blue extravasation

Determination of Evans blue extravasation was based on a previous method [16] with minor modifications. After reperfusion, 0.1 ml of 4% Evans blue (Urchem, Shanghai, PR China) in 0.9% saline was intravenously administered. Twenty-three hours after I/R, rats were anesthetized with chloral hydrate (350 mg/kg, *i*.*p*.), then perfused with 20 ml 10 U/ml heparinized saline to wash out the blood, the brain was then isolated, weighed and homogenized in 50% solution of trichloroacetic acid. After centrifugation at 400 × g for 20 minutes, the supernatant was spectrophotometrically measured at 595 nm. Cerebral Evans blue was quantified as micrograms of dye per gram of wet weight.

### NF-κB binding assay

SH-SY5Y cells (5 × 10^6^) were pre-incubated with RA 9 μM for 22 h, then incubated with TNF-α (20 ng/ml) for 1 h or 2 h, then washed once with phosphate-buffered saline (PBS), scraped cells into 1 mL cold PBS, and pelleted by centrifugation. Nuclear extracts were prepared as described previously [17]. The DNA binding activity of NF-κB (p50/p65) was determined using an ELISA kit (Solar Biology Technology Company, Shanghai, PR China).

### Western blots analysis

For the experiment of TNF-α stimulated SH-SY5Y cell lines *in vitro*, SH-SY5Y cells (5 × 10^6^) were pre-incubated with RA (9 μM) or HMGB1 inhibitor, glycyrrhizin (100 μM) for 120 minutes, then incubated with TNF-α (20 ng/ml) for 30 minutes and cultured in a CO_2_ incubator for 12 h. Cells were washed twice with ice cold PBS on ice and lysed in NP40 lysis buffer (Biosource, Camarillo, CA, USA) (50 mM Tris, pH 7.4, 250 mM NaCl, 5 mM EDTA, 50 mM NaF, 1 mM Na_3_VO_4_, 1% NP-40 and 0.02% NaN_3_) supplemented with 1 mM PMSF and 1 × protease inhibitor cocktail (Sigma, Saint Louis, MO, USA).

For Western blot analysis of collected brain tissues, the tissues were defrosted and immersed in ice-cold NP40 lysis buffer. Equal amounts of cell protein (50 μg) were separated by SDS-PAGE and analyzed by Western blot using specific antibodies to HMGB1, IκB, phosphor-IκB-α, phosphor-NF-κB and proliferating cell nuclear antigen (PCNA, loading control). All antibodies were purchased from Beijing Biosynthesis Biotechnology Company (Beijing, PR China). Optical densities of the bands were scanned and quantified with a Gel Doc 2000 (Bio-Rad laboratories, Milan, Italy). Data were normalized against those of the corresponding PCNA bands. Results were expressed as fold increase over control or sham group.

### Myeloperoxidase (MPO) activity assay

The remaining slices of the brain, not used for Western blot analysis, were used to determine MPO activity as described previously [18]. For each individual rat, the ischemic and non-ischemic hemispheres (from the six slices) were pooled separately for the assay, and wet weight was then recorded. The tissues were homogenized (1: 20, wt/vol) in 5 mmol/L phosphate buffer (pH 6, 4°C) and centrifuged at 30,000 g for 30 minutes (4°C). MPO activity was determined according to the kit instructions (Xitang Biology Technology Company, Shanghai, PR China). MPO activity for each tissue sample is normalized on the basis of grams per wet weight of tissue.

### NeuN staining

The left hemisphere of the brain was cut coronally into three blocks from the level of the optic chiasm and the infundibular stem of the hypophysis. The middle block was further cut into three sub-blocks. The middle sub-block (0.1 × 0.1 cm^2^) was embedded in paraffin. NeuN-immunolabeling was carried out according to the previous method [19]. NeuN-immunopositive cells were counted in three randomly selected fields (400 ×). The number of NeuN-immunopositive cells was calculated by averaging the three counts.

### Statistical analysis

Neurological deficit scores between groups were analyzed using a non-parametric test. Quantitative data from the experiments were expressed as mean ± standard deviation (S.D.); significance was determined by one-way analysis of variance (ANOVA) followed by Dunnett's test (SPSS 16.0. IBM Company,Chicago, USA). In all cases, differences were considered significant if *P* <0.05.

## Results

### Effects of RA on cultured SH-SY5Y cells against oxygen-glucose deprivation-induced cytotoxicity and apoptosis

As estimated by MTT assay, cell viability was markedly decreased after OGD for 3 h followed by 12 h incubation with neurobasal medium (Table 1). However, when cells were incubated with RA, the cytotoxicity was significantly attenuated in a concentration-dependent manner, as shown in Table 1. To further investigate the protective effect of RA, LDH leakage rate was estimated, a significant increase of LDH leakage rate was observed after oxygen-glucose deprivation. Incubation with various concentrations of RA significantly inhibited oxygen-glucose deprivation-induced LDH release in a concentration-dependent manner.

**Table 1:** **Effects of RA on the viability and LDH leakage in SH**-**SY5Y cells exposed to oxygen**-**glucose deprivation**

**Groups**	**OGD**	**Content (μM)**	**Cell viability (%)**	**LDH leakage (%)**
Normal	_	—	100.0 ± 4.7	4.1 ± 1.2
Control	+	—	52.0 ± 6.8^#^	24.7 ± 4.2^#^
RA	+	1	59.2 ± 7.3	21.9 ± 2.8
	+	3	64.2 ± 5.8^*^	19.6 ± 3.5^*^
	+	9	69.0 ± 9.1^**^	16.5 ± 2.1^**^
	+	27	72.8 ± 7.4^**^	14.0 ± 3.1^**^
	+	81	73.5 ± 8.3^**^	13.3 ± 1.7^**^

After 3 h oxygen-glucose deprivation (OGD) followed by 12 h incubation with RA, cell viability and LDH leakage were assessed. RA indicated Rosmarinic acid. OGD, oxygen-glucose deprivation. Values are mean ± S.D. (n = 6). Significance was determined by one-way ANOVA followed by Dunnett's test. ^#^*P* <0.01 versus normal group; ^*^P <0.05, ^**^P <0.01 versus Control group.

**Figure 1:**
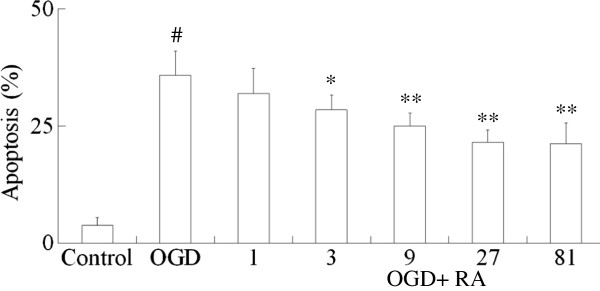
**Effects of RA on apoptosis of cultured SH**-**SY5Y cells.** After 3 h oxygen-glucose deprivation (OGD) followed by 12 h incubation with RA, cell apoptosis was evaluated by flow cytometric analysis. RA indicates rosmarinic acid. Values are mean ± S.D. (n = 6). Significance was determined by one-way ANOVA followed by Dunnett's test. ^#^*P* <0.01 *vs* Control group; ^*^P <0.05, ^**^P <0.01 *vs* OGD group.

Apoptotic cells were estimated by flow cytometric analysis of annexin-V and propidium iodide-labeling cells, as shown in Figure 1. The normal SH-SY5Y cell apoptosis rate was only 3.6 ± 1.5%, after oxygen-glucose deprivation for 3 h followed by 12 h incubation with neurobasal medium, the apoptosis of oxygen-glucose deprivation was increased to 35.8%. Incubation with RA (3 to 27 μM) for 12 h arrested the apoptosis in a concentration-dependent manner.

### Effects of RA on NF-κB activation and HMGB1 expression

**Figure 2:**
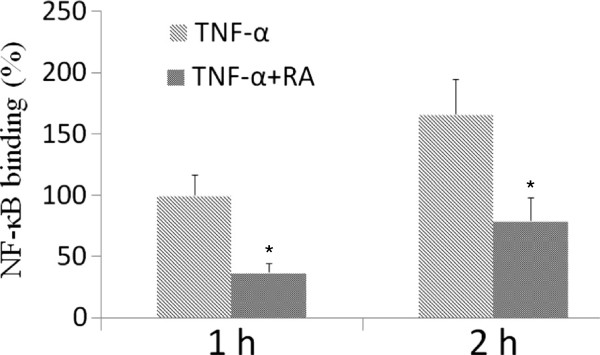
**Effect of RA on TNF-α-induced increase of p50**/**p65 binding to DNA.** SH-SY5Y cells were pre-incubated with RA (9 μM) for 22 h, then incubated with TNF-α (20 ng/ml) for 2 h. The DNA binding activity of NF-κB (p50/p65) was determined using an ELISA kit. RA indicates rosmarinic acid. Results are expressed as fold increase over control, n = 5. ^*^P <0.01 *vs*. TNF-α-induced group. ^#^*P* <0.01 *vs*. control group. Significance was determined by one-way analysis of ANOVA followed by Dunnett’s test.

The NF-κB pathway plays a critical role in the secretion of cytokines. The quantity of p50 and p65 was measured in the nucleus. Stimulation with TNF-α led to a robust activation of the NF-κB transcription factor p50/p65. This activation was partially blocked by RA, as shown in Figure 2.

**Figure 3:**
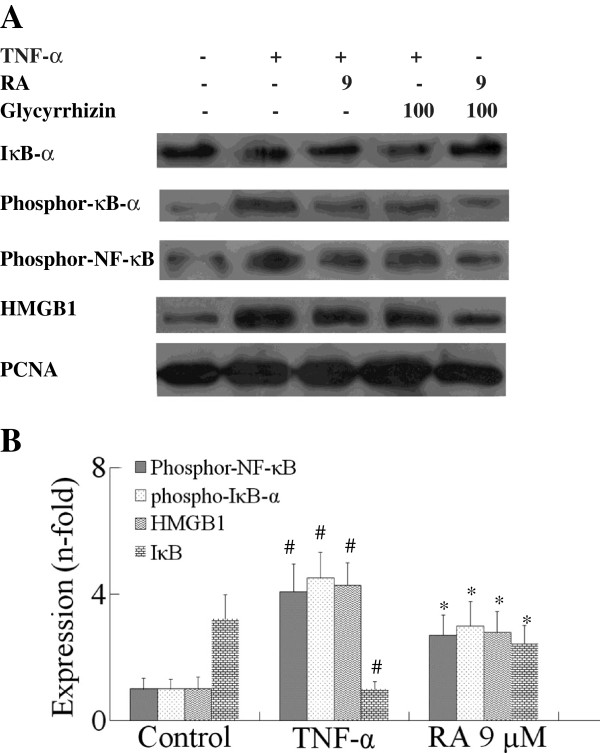
**Effects of RA on TNF-α-induced HMGB1 and NF-κB activation.** SH-SY5Y cells were pre-incubated with HMGB1 inhibitor, glycyrrhizin (100 μM) or RA (9 μM) for 120 minutes and then incubated with TNF-α (20 ng/ml) for 30 minutes. HMGB1, IκB, phospho-IκB-α and phospho-NF-κB expression were analyzed by Western blotting. RA indicates rosmarinic acid. Results are expressed as fold increase over control, n = 5. ^*^P <0.05, ^**^P <0.01 *vs*. TNF-α-induced group. ^#^*P* <0.01 *vs*. control group. Significance was determined by one-way analysis of ANOVA followed by Dunnett’s test.

The IκB kinase system as another activation agent of NF-κB was also examined. Stimulation with TNF-α resulted in a marked degradation of IκB. This degradation was inhibited by RA (Figure 3). In addition, the phosphorylation of p-IκB was increased by TNF-α, and it was also inhibited by RA.

Phosphor-NF-κB expression was low in SH-SY5Y cells. However, phosphor-NF-κB expression was significantly increased after TNF-α 20 ng/ml stimulated SH-SY5Y cells for 120 minutes. We compared the effect of RA on the TNF-α-induced activation of phosphor-NF-κB and HMGB1 expression level in the SH-SY5Y cells to that of a selective HMGB1 inhibitor, glycyrrhizin. The results of Figure 3 showed that pretreatment of SH-SY5Y cells with glycyrrhizin (100 μM) for 120 minutes blocked the TNF-α-induced HMGB1 expression and reduced phosphorylation of NF-κB. Pretreatment of SH-SY5Y cells with RA 9 μM blocked TNF-α-induced NF-κB phosphorylation, and reduced HMGB1 expression, as shown in Figure 3.

### Effects of RA on brain infarct volume, brain water content and neurological deficit scores in cerebral I/R rats

Cerebral I/R injury leads to severe behavioral disturbance and histological changes in diabetic MACO rats, ischemic regions appeared white while non-ischemic region appeared red. Compared with the diabetic animals, neurological deficit scores, the white regions and brain water content were significantly higher in diabetic cerebral I/R rats.

The results of the dose–response study indicated that RA at doses of 25, 50, 100 and 200 mg/kg decreased neurological deficit scores, reduced cerebral infarct volume and brain water content dose-dependently, did not reduce blood glucose levels, as shown in Table 2.

**Table 2:** **Effects of RA on ischemia**-**reperfused diabetic rats**: **a dose**–**response study**

**Group**	**Survival (# rats)**	**Neurological scores (median/range)**	**Blood glucose (mM)**	**Infarct volume (%)**	**Brain water content (%)**
Control	10/10	—	5.2 ± 0.6	—	76.8 ± 0.3
Diabetic	10/10	—	19.1 ± 2.3	—	77.0 ± 0.4
Vehicle	8/10	4/2^#^	19.8 ± 2.5	28.4 ± 5.3^#^	79.5 ± 0.4^#^
RA 12.5	9/10	3/3	19.6 ± 2.6	24.5 ± 5.4	79.3 ± 0.4
RA 25	9/10	3/4^*^	19.5 ± 2.4	21.8 ± 5.7^*^	79.0 ± 0.5^*^
RA 50	10/10	2/3^**^	20.1 ± 2.7	19.7 ± 5.2^**^	78.9 ± 0.3^**^
RA 100	10/10	2/3^**^	19.7 ± 2.6	16.5 ± 4.4^**^	78.7 ± 0.4^**^
RA 200	10/10	2/3^**^	19.6 ± 2.8	15.5 ± 3.2^**^	78.6 ± 0.5^**^

Data are means ± SD, with n = 10 for each group. RA at doses ranging from 12.5 to 200 mg/kg, was administered intravenously 30 minutes after cerebral I/R. RA indicates rosmarinic acid, Control indicates non-diabetic rats. ^#^*P* <0.01 *vs*. Diabetic group; ^*^P <0.05, ^**^P <0.01 *vs* Vehicle-treated group. Cerebral infarct volume and brain water content between groups were compared using one-way ANOVA followed by Dunnett's test. Neurological scores between groups were compared using a nonparametric test.

The results of the therapeutic time-window study indicated that RA at a dose of 50 mg/kg decreased neurological deficit scores, reduced brain infarct volume and brain water content even with delayed administration 1 h, 3 h and 5 h after reperfusion. It was obvious that earlier administration of RA brought more therapeutic benefits, as shown in Table 3.

**Table 3:** **Effects of RA on survival**, **neurological scores**, **infarct volume and brain water content in ischemia**-**reperfused diabetic rats**: **a therapeutic time**-**window study**

**Group**		**Survival (# rats)**	**Neurological scores (median/range)**	**Infarct volume (%)**	**Brain water content (%)**
**Diabetic**		10/10	—	—	77.0 ± 0.5
**Vehicle-treated**		8/10	4/2	27.7 ± 4.7	79.6 ± 0.4^#^
	1 h	10/10	2/3^**^	19.0 ± 4.4^**^	78.8 ± 0.4^**^
**RA**	3 h	10/10	2/3^*^	21.5 ± 4.5^**^	79.0 ± 0.4^*^
	5 h	9/10	3/4^*^	22.8 ± 2.8^*^	79.1 ± 0.4^*^
	7 h	9/10	3/4	24.1 ± 4.0	79.3 ± 0.4

Data are means ± SD, with n = 10 for each group. RA was administered at a dose of 50 mg/kg at 1 h, 3 h, 5 h and 7 h after cerebral I/R. RA indicates rosmarinic acid. Data are means ± SD. ^#^*P* <0.01 *vs*. diabetic animals; ^*^P <0.05, ^**^P <0.01 vs vehicle-treated group. Cerebral infarct volumes of different groups were compared using one-way ANOVA followed by Dunnett's test. Neurological deficit scores between groups were compared using a nonparametric test.

**Figure 4:**
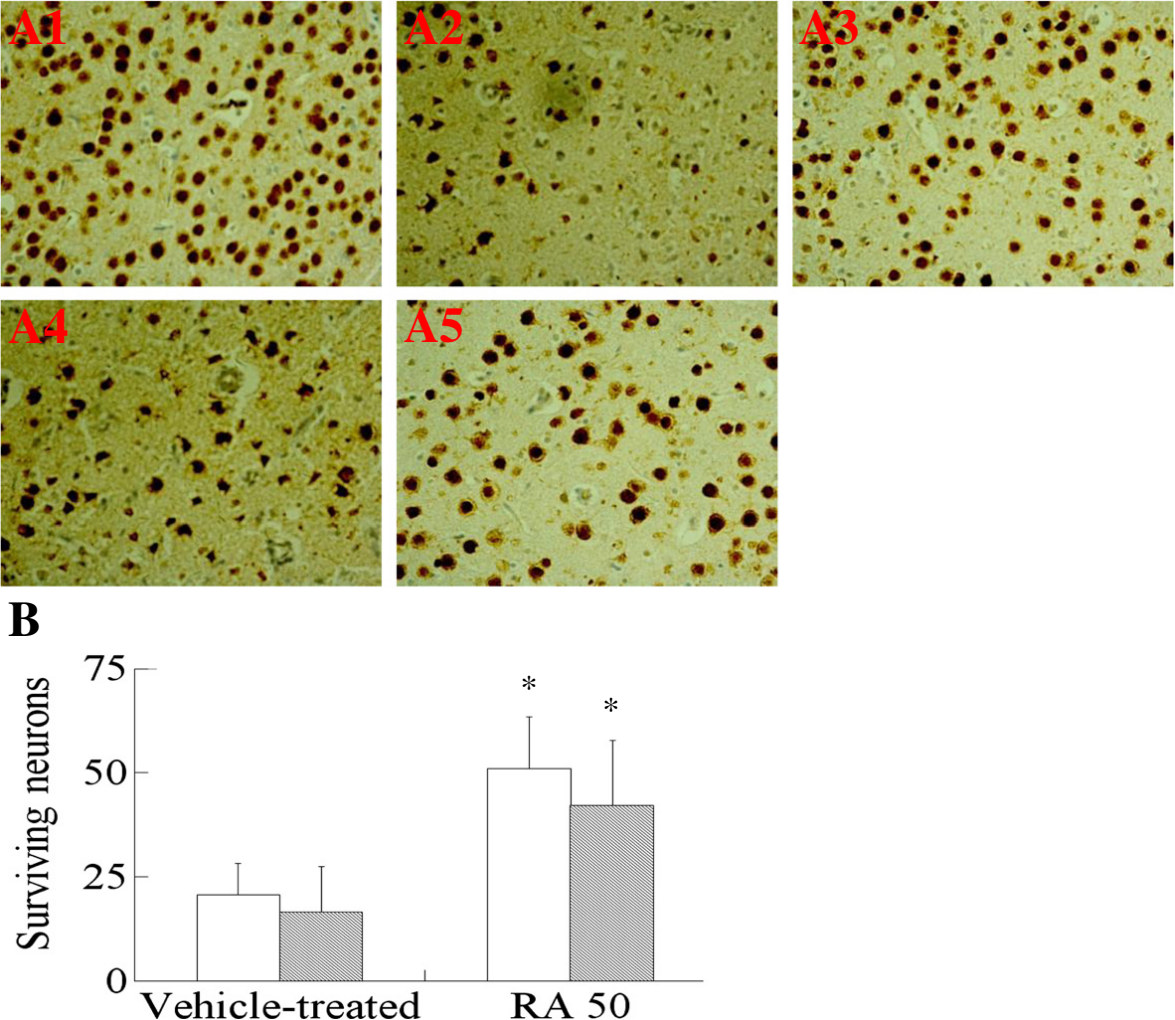
**Effect of RA on neurological damage 24 h and 14 days in diabetic cerebral ischemia**-**reperfusion (I/R) rats. A**: Effect of RA on cerebral pathological damage after cerebral I/R in diabetic rats. Sections were stained with NeuN after fixation. A1: diabetic group; A2: Vehicle-treated group (24 h); A3: RA 50 mg/kg group; A4: Vehicle-treated group (14th day); A5: RA, 50 mg/kg, group (14th day). **B**: Effect of RA on the NeuN-immunopositive neurons after cerebral I/R in diabetic rats. RA indicates rosmarinic acid. Results were averaged and expressed as the number of NeuN-immunopositive neurons per section. Data are means ± SD, n = 5. ^*^P <0.01 *vs* Vehicle-treated group. Significance was determined by one-way ANOVA followed by Dunnett’s test.

The results of the long-term study indicated that RA at a dose of 50 mg/kg significantly decreased neurological deficit scores and reduced cerebral infarction 14 days after I/R, as shown in Table 4. The results of NeuN immunolabeling indicated that this is due to a significantly increased number of surviving neurons 24 h and 14 days after cerebral I/R, as shown in Figure 4. Thus, it was clear that early treatment with RA provided long-term benefits for the neuronal functional recovery after cerebral I/R.

**Table 4:** **Effects of RA on survival**, **neurological scores**, **blood glucose and infarct volume in ischemia**-**reperfused rats**: **a long**-**term study**

**Group**	**Time (days)**	**Survival (# rats)**	**Neurological scores (median/range)**	**Blood glucose (mM)**	**Infarct volume (%)**
**Vehicle**	3	12/15	4/3	20.3 ± 3.1	30.3 ± 5.1
	7	12/15	3/4	20.5 ± 3.2	
	14	12/15	3/4	20.7 ± 3.4	
**RA50**	3	14/15	2/4^**^	20.5 ± 2.9	
	7	14/15	2/4^*^	20.4 ± 3.1	
	14	14/15	2/4^*^	20.7 ± 3.2	13.9 ± 4.7^**^

RA was administered at a dose of 50 mg/kg as a single intravenous bolus injection 30 minutes after cerebral I/R. RA indicates rosmarinic acid. Data are means ± SD, each group contains 15 rats. ^*^P <0.05, ^**^P <0.01 *vs* vehicle-treated group. Cerebral infarct volumes of different groups were compared using one-way ANOVA followed by Dunnett's test. Neurological deficit scores between groups were compared using a nonparametric test.

### Effects of RA on cerebral Evans blue extravasation in I/R rats

Evans blue extravasation was used to assess of BBB breakdown after cerebral ischemia in diabetic rats. Cerebral I/R injury led to higher Evans blue extravasation compared with the diabetic rats. The results clearly showed that Evans blue extravasation was significantly attenuated by RA treatment, as shown in Table 5.

**Table 5:** **Effect of RA on cerebral Evans blue extravasation and MPO activity in ischemia**-**reperfused rats**

**Group**	**Dose (mg/kg)**	**Evans blue extravasation (μg/g wet weight)**	**MPO activity (U/g wet weight)**
Control	—	0.59 ± 0.13	0.02 ± 0.01
Diabetic	—	0.67 ± 0.18	0.03 ± 0.01
Vehicle	—	3.93 ± 0.62^#^	0.18 ± 0.04^#^
RA	50	1.73 ± 0.42^*^	0.09 ± 0.02^*^

Data are means ± SD, with n = 5 for each group. ^#^*P* <0.01 *vs*. diabetic animals; ^*^P <0.01 *vs* vehicle-treated animals. Significance was determined by one-way ANOVA followed by Dunnett's test.

### Effects of RA on cerebral MPO activity, NF-κB activation and HMGB1 expression in I/R rats

**Figure 5:**
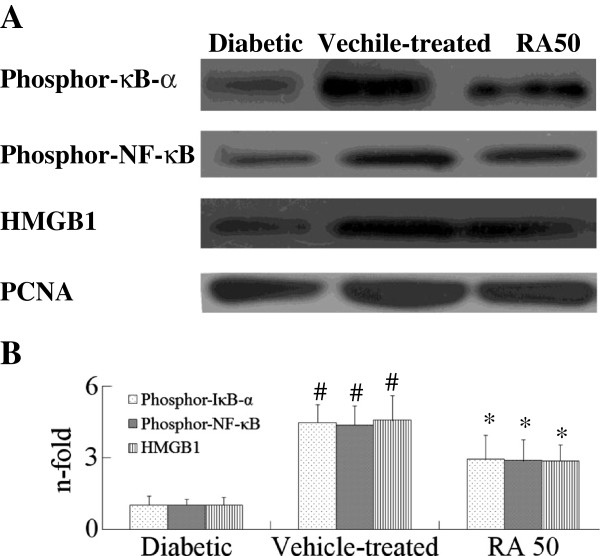
**Effects of RA on phosphor-NF-κB, phosphor-IκB-α and HMGB1 in cerebral I/R rats by western blots analysis 24 h after I/R.** Diabetic rats were subjected to middle cerebral artery occlusion (MCAO) and reperfusion (I/R) for 23 h. Total protein extracts were prepared and assayed for phosphor-NF-κB, phosphor-IκB-α and HMGB1 by Western blot analysis, and blots were normalized to PCNA expression. RA indicates rosmarinic acid. Results are expressed as fold increase over sham group, n = 5. ^*^P <0.01 *vs*. Vehicle-treated group. ^#^*P* <0.01 *vs*. Diabetic group. Significance was determined by one-way analysis of ANOVA followed by Dunnett’s test.

In order to investigate the molecular mechanism of RA, cerebral MPO activity, NF-κB activation and HMGB1 expression were examined. Cerebral MPO activity increased in rats after cerebral I/R 24 h, but it was very weakly presented in the diabetic animal, as shown in Table 5. RA treatment reduced MPO activity in cerebral tissue. Phosphorylation of IκB-α and NF-κB both increased in occurrence in rats after cerebral I/R, HMGB1 expression also increased, but they were very weakly presented in the diabetic animals, as shown in Figure 5. However, RA treatment not only reduced HMGB1 expression, but also decreased phosphorylated IκB-α and NF-κB levels. These data indicated that RA inhibited inflammation response by reducing MPO activity, blocking NF-κB activation and HMGB1 expression in diabetic cerebral I/R rats.

## Discussion

The present study showed OGD for 3 h followed by 12 h incubation could simulate the brain hypoxia. The neuronal apoptosis could be activated after treatment with OGD for 3 h followed by 12 h incubation. The results showed RA noticeably reduced apoptosis and necrosis, increased cell survival and decreased LDH leakage rate in cultured SH-SY5Y) cells. In *in vivo* study, we observed RA significantly improved brain injury and did not reduce blood glucose in diabetic rats subjected to cerebral I/R challenge; this meant RA has immediate neuroprotective effect, not by reducing blood glucose.

Infarct size and brain edema are the two important indicators of the pathophysiology used to evaluate the efficacy of cerebral ischemia [20]. Our results (Table 2) revealed that RA treatment produced significant reduction of cerebral infarct volume and brain water content in diabetic cerebral I/R rats. Neuronal degeneration and necrosis have been found to be correlated to deficits in behavioral disturbance, and behavioral assessment may reveal the effectiveness of a neuroprotective drug [21]. The present study showed that neurological scores were reduced by treatment with RA (Table 2). NeuN is a sensitive marker for injured neurons early after ischemic challenge [22]. Our data (Figure 4) demonstrated that RA 50 mg/kg attenuated the decrease of NeuN-immunopositive neurons in ischemic cerebral cortex 24 h and 14 days after I/R. This indicated that RA has potentially beneficial effects in the treatment of cerebral ischemia.

A recent update of the Stroke Therapy Academic Industry Roundtable Preclinical Recommendations [23] lists three potential reasons for failures in translating efficacious preclinical findings into successful clinical trial outcomes; (i) a very tight dose range with a steep dose–response curve; (ii) inadequate investigation of the therapeutic time-window for initiation of treatment and (iii) differences in the observation period between animal models and clinical trials. Consequently, we investigated the dose–response, therapeutic time window and the long-term efficacy of RA in cerebral I/R rats. Our data demonstrated that RA exerted potent and long-term neuroprotective effects with an appropriate dose–response curve and a favorable therapeutic time-window in the model of diabetic cerebral I/R.

Stroke triggers an inflammatory reaction that progresses for hours after the onset of a stroke, and this inflammation plays a central role in the pathogenesis of neuronal injury in ischemic stroke, and especially in diabetic stroke [24]. Inflammatory reactions contribute to the late stages of ischemic injury and to worsened neurological outcome through multiple mechanisms. The present study indicated that RA had anti-inflammatory effects (Table 5, Figures 3 and 4). Treatment with RA especially provided long-term benefits for neuronal functional recovery after cerebral I/R (Table 4). This suggested that the neuroprotective effects of RA might be due to blocking the inflammatory response.

MPO is considered an index of neutrophil infiltration, and highly expressed cerebral ischemia after 24 h. It showed that a significant correlation between neutrophil infiltration and infarct formation exists in a model of cerebral ischemia [25]. Our results showed that RA reduced MPO activity in diabetic ischemic cerebral tissue. This suggested that the neuroprotective effects of RA might be due to blocking neutrophil infiltration (Table 5).

BBB permeability is significantly increased in diabetes due to diabetes-induced damage to the BBB function and/or due to the immature nature of the newly formed vessel [26]. BBB breakdown also occur in early phases (within 24 h) of cerebral ischemia [27]. Our data demonstrated that RA improved diabetic cerebral I/R injury by attenuating BBB breakdown.

It is well established that NF-κB activation is associated with the phosphorylation of IκB-α and NF-κB in diabetic ischemic cerebral tissue. Reduction of NF-κB activation can protect brain from activation of NF-κB-dependent genes [28]. Our previous study showed that RA blocked NF-κB activation in the model of sepsis [11]. HMGB1 is a novel player in the ischemic brain [29]. Diabetes significantly increased serum HMGB level and induced worse functional outcome after stroke compared to non-streptozotocin (WT) MCAO rats [30]. Diabetes exacerbates systemic inflammation as evidenced by higher serum HMGB1 in the rat systemic inflammation model [31]. All those indicated HMGB-1 played a key role in diabetic stroke. HMGB-1 signaling promotes the chemotaxis and production of cytokines in a process that involves the activation of NF-κB [32]; inhibitors of NF-κB kinases alpha and beta are both essential for high mobility group box 1-mediated chemotaxis [33]. Down-regulation of HMGB1 and NF-κB expression protected rat brains against focal ischemia. Suppression of the release of HMGB1 in astrocytes leads to the attenuation of neuroinflammation, preventing the necrosis of ischemic astrocytes and NF-κB expression [34]. HMGB1 recruits various transcription factors, including NF-κB [35,36]. Inhibition of the up-regulation of HMGB1 and NF-κB at the early stage brings great benefits to cerebral ischemia. Based on the above research, we explored the anti-inflammatory properties of RA in diabetic cerebral ischemia, and further studied the potential mechanisms. The up-regulation of HMGB1 and NF-κB were significantly suppressed by RA. These results suggested that suppressing the expressions of HMGB1 and NF-κB participated in the neuroprotection of RA against diabetic cerebral ischemic damage. Therefore, we believed that the protective effects of RA might be due to the suppression of the inflammatory cascades through HMGB1 dependent NF-κB signaling pathway.

In summary, the results of the current study suggest that RA exhibit significant neuroprotective effects during diabetic cerebral I/R injury, including attenuation of BBB breakdown, a decrease of infarct volume, alleviation of cerebral damage, reduction of HMGB1 expression, phosphorylated IκB-α and NF-κB protein expression in ischemic brain tissue. These effects of RA were correlated with inhibition of the inflammatory response. These findings point to a therapeutic potential for RA as a useful anti-inflammatory lead compound in early diabetic cerebral I/R injury.

## Abbreviations

BBB: Blood–brain barrier; BSS: Balanced salt solution; DM: Diabetes mellitus; DMEM: Dulbecco’s modified Eagle’s medium; FBS: Fetal bovine serum; FITC: Fluorescein isothiocyanate; HMGB1: High-mobility group box1; i.p.: Intraperitoneal; I/R: Ischemia and reperfusion; LDH: Lactic dehydrogenase; MACO: Middle cerebral artery occlusion; MAP: Mean arterial pressure; MCAO: Middle cerebral artery occlusion; MPO: Myeloperoxidase; MTT: 3-(4, 5)-dimethylthiahiazo (−z-y1)-3, 5-di-phenytetrazoliumromide; NF-κB: Nuclear transcription factor κB; OGD: Oxygen-glucose deprivation; PBS: Phosphate-buffered saline; RA: Rosmarinic acid; STZ: Streptozotocin; TNF-α: Tumor necrosis factor-α; TTC: Tetrazolium chloride; WT: Wild type.

## Competing interests

The authors declare that they have no competing interests.

## Authors’ contributions

WJ and CL contributed to the design of the study. HL contributed to NeuN staining. WJ, ZK, HL and XY contributed to animal experiments and cell culture. All authors read the manuscript, studied it critically for its intellectual content and approved the final draft. All authors read and approved the final manuscript.
